# Supplementary material for: Unveiling Nilaparvata lugens Stål Genes Defining Compatible and Incompatible Interactions with Rice through Transcriptome Analysis and Gene Silencing
Source: Curr Issues Mol Biol. 2023 Aug 16;45(8):6790–803. doi: 10.3390/cimb45080429 (PMC10453277; doi:10.3390/cimb45080429)
Supplement: Supplementary file 1 [file cimb-45-00429-s001.zip › Table S1.pdf]

Supplementary Table S1. List of primers used to evaluate the expression of the selected DEGs via RT-qPCR.

**Gene ID or name**	**Forward sequence (5′-3′)**	**Reverse sequence (5′-3′)**
LOC111058179	CTCGCGAACACTTCCGAAAC	GATGGCCACGTTGCTTGAAC
LOC111056854	TTGAAGGACTTACCGGCTCG	GTGGAGTCTGTGAACGGTCC
LOC111064160	AGAACCTCGGCATCCCTGAT	GGCTCAACATTTCCTCGCAC
LOC111064061	ATGTGTGCTTCTGAGAGGGC	CAAGGAGCTTCACTGGAGGG
LOC111060644	AGGAAAACAAGGGCTACCTCG	ATGGTTCCGTTGACAGGGTC
LOC111046236	CGTGAGATACGAGGGAGAAGG	CGGCATACTTGGGTGCAGAATA
LOC111046246	GTCATCAAATACGCACCCGC	TCGAATGATGTCCGCTGCTT
LOC111059963	TGTTGCGTTCACGGACTCG	GGCCAAGTCTTCATGTCCTGT
LOC111063850	AAAACGTGACAACAGACGTG	CCAGGTGAACCCGACATGAA
LOC111052989	TGCTGCATTTGTGAGGCAAC	ATCACGTTGAACCCGACCTC
RPS15	TAAAAATGGCAGACGAAGAGCCCAA	TTCCACGGTTGAAACGTCTGCG
Tub	ACTCGTTCGGAGGAGGCACC	GTTCCAGGGTGGTGTGGGTGGT
