# Supplementary material for: Unveiling Nilaparvata lugens Stål Genes Defining Compatible and Incompatible Interactions with Rice through Transcriptome Analysis and Gene Silencing
Source: Curr Issues Mol Biol. 2023 Aug 16;45(8):6790–803. doi: 10.3390/cimb45080429 (PMC10453277; doi:10.3390/cimb45080429)
Supplement: Supplementary file 1 [file cimb-45-00429-s001.zip › Table S2.pdf]

**Supplementary Table S2**. List of primers used to synthesize dsRNAs for the selected DEGs.

| **Gene name** | **Forward sequence (5′-3′)** | **Reverse sequence (5′-3′)** |
|---|---|---|
| *NlCP1* | TCCACAAGAAACCACGCCTT | GAGTAGCCGGATGAAGCGTA |
| *NlCYP320a1* | CCTGAGGCTGAAGGACACTG | ATCCAACTCGGCGACCTTTT |
| *NlCarE6* | GCTTGGGACATCAAGGGACA | TCTCTTCGGCATATTCGGGC |
| *NlTret1* | CTCGCGAACACTTCCGAAAC | TAGAAGGCGGAGCGTGTTTT |
| *GFP* | CACAAGTTCAGCGTGTCCG | GTTCACCTTGATGCCGTTCT |
