# Supplementary figures and images for: Unveiling Nilaparvata lugens Stål Genes Defining Compatible and Incompatible Interactions with Rice through Transcriptome Analysis and Gene Silencing
Source: Curr Issues Mol Biol. 2023 Aug 16;45(8):6790–803. doi: 10.3390/cimb45080429 (PMC10453277; doi:10.3390/cimb45080429)

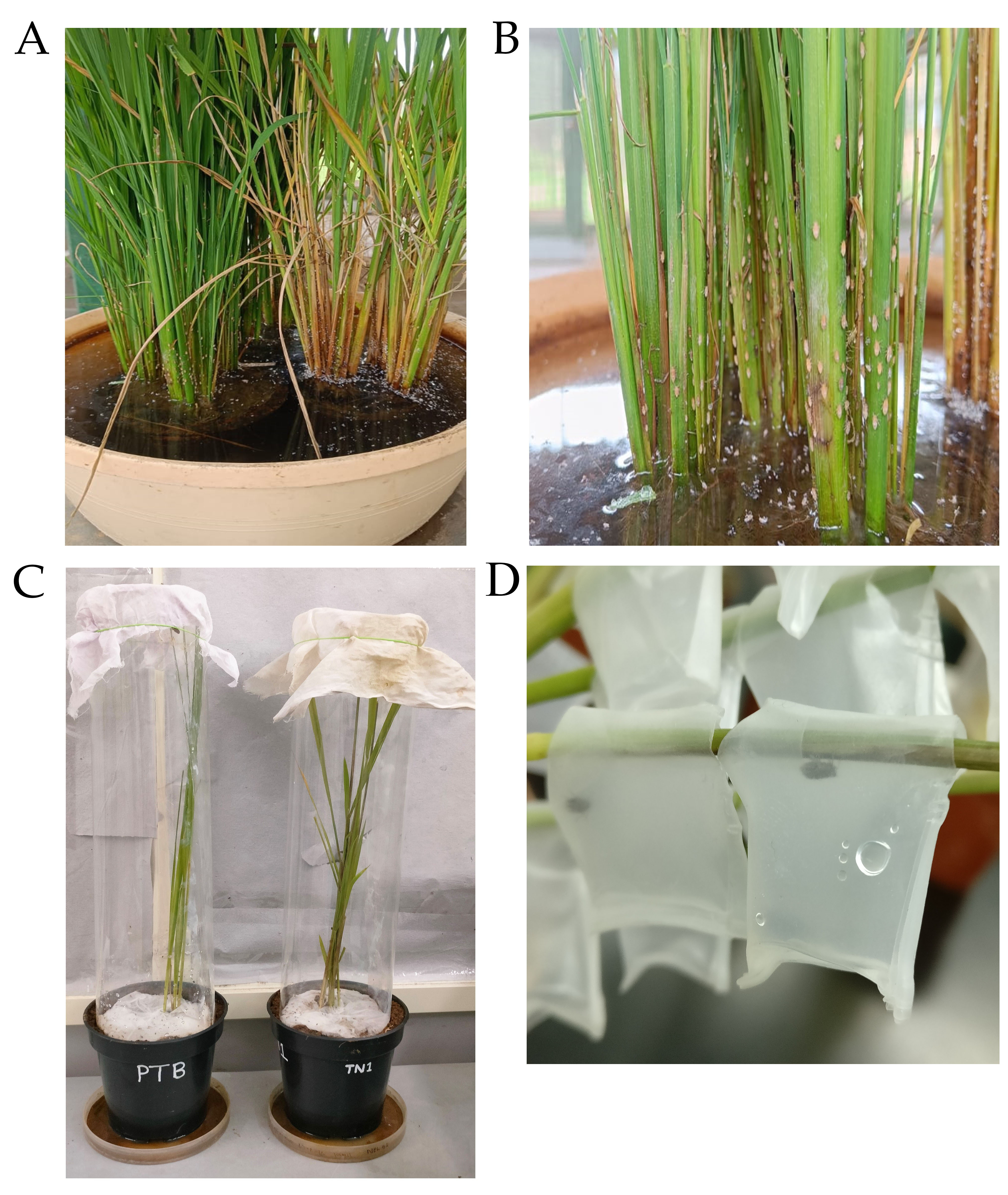

Supplement: Supplementary file 1 [file cimb-45-00429-s001.zip › Figure S1.jpg]
